# Modeling Social Network Topologies in Elementary Schools

**DOI:** 10.1371/journal.pone.0055371

**Published:** 2013-02-07

**Authors:** Rodrigo Huerta-Quintanilla, Efrain Canto-Lugo, Dolores Viga-de Alva

**Affiliations:** 1 Departamento de Física Aplicada, Centro de Investigación y de Estudios Avanzados del Instituto Politécnico Nacional, Unidad Mérida, Mérida, Yucatán, México; 2 Departamento de Ecología Humana, Centro de Investigación y de Estudios Avanzados del Instituto Politécnico Nacional, Unidad Mérida, Mérida, Yucatán, México; Université de Lausanne, Switzerland

## Abstract

Complex networks are used to describe interactions in many real world systems, including economic, biological and social systems. An analysis was done of inter-student friendship, enmity and kinship relationships at three elementary schools by building social networks of these relationships and studying their properties. Friendship network measurements were similar between schools and produced a Poisson topology with a high clustering index. Enmity network measurements were also similar between schools and produced a power law topology. Spatial confinement and the sense of belonging to a social group played vital roles in shaping these networks. Two models were developed which generate complex friendship and enmity networks that reproduce the properties observed at the three studied elementary schools.

## Introduction

Complex networks are widely applied in disciplines as varied as economics [1], biology [2], information technology [3] and sociology [4], [5]. Further development of complex networks theory is therefore a vital research area, with recent efforts focusing on measurements [6], topologies [7], [8] and the way data is disseminated through them [9].

Complex networks are a tool for modeling systems in which elements interrelate. Social networks are systems that describe phenomena in which individuals interact within a society (e.g. people, companies, etc.); nodes represent individuals and links represent the social relationships between them. Recent research has focused on the patterns of face-to-face interaction dynamics. In one study, radio frequency identification devices were used to calculate the proximity and duration of interpersonal interactions, and thus create social networks to understand community behavior and apply diffusion processes for infectious diseases and information [10]. Using the same technology, studies have been done in high schools [11] and elementary schools [12] of the mixing patterns of students in a school environment that describe social network’s temporal evolution and apply infectious disease diffusion processes to identify high-risk situations and establish vaccination strategies.

When studying data dissemination within a social system, an understanding is needed of the network topology that models the interactions produced within it. To this end, the present study objective was to evaluate the properties of friendship and enmity networks representing interactions between elementary school students and develop models that reproduce them. This will facilitate future research into problems such as scholastic performance, disease transmission and evolution of the cultural environment, among other important phenomena occurring in schools which could benefit from the formalism of complex networks [13]–[15].

We describe the methodology used to collect the data and generate the databases used in developing the networks. These data have certain characteristics that are not reproduced by classic models of complex network theory. The tests used to analyze friendship networks are described in section ‘Friendship Networks Analysis’ and implementation of the proposed model is described in section ‘Friendship Network Model’, while the enmity networks are addressed in section ‘Enmity Network Analysis’ and the proposed descriptive model in section ‘Enmity Network Model’. Promising future research emphases are proposed.

## Methodology

The methodology used in this research was approved by the Bioethics Committee for Research in Human Beings of the Centro de Investigación y de Estudios Avanzados del IPN. We obtained written consent from the guardians of the children who participated in this study. Also, all data was analyzed anonymously once these arrived to researchers.

No empirical data were available for analysis, so we designed and applied an instrument to 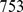 students at three elementary schools. This confidential, mixed questionnaire [16] consisted of twelve questions, six for general student data and six for data on friendship, enmity and kinship relationships between students at the same school. To avoid conflict or misunderstanding, the term ‘enmity’ was replaced by ‘non-affective relationships’ in the questionnaire. The instrument was applied by three qualified survey takers to groups of ten students at a time. A pilot test was run previously at one of the studied schools to identify any problems and confirm questionnaire item clarity.

The instrument was applied at three elementary schools where first through sixth grades are taught:

1. School1: rural, 108 students in 6 classrooms.
2. School2: rural, 226 students in 9 classrooms.
3. School3: urban, 419 students in 12 classrooms.

One classroom at School1 contained two groups, 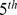 and 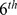 grades, although each group engaged in separate activities.

After collection, the data were used to build three adjacency matrices: 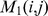, 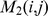 and 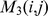, where 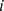 and 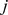 are students. These were categorized as follows:


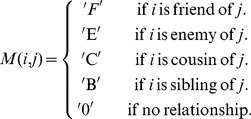
(1)

Consistency within the data was attained by applying logic rules and research hypotheses:

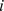
 and 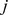 are siblings 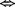 at least one says they are the sibling of the other and they have the same surnames.
2. If 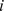 is sibling to 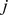 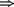 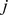 is sibling to 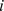.
3. If 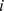 is sibling to 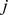 and 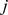 is sibling to 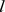 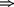 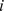 is sibling to 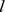.

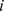
 and 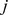 are cousins 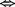 at least one says they are the cousin of the other and they have a surname in common.
5. If 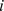 is cousin to 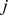 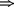 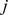 is cousin to 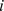.
6. If 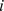 is cousin to 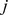 and 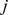 is sibling to 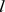 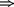 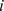 is cousin to 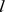.
7. Friendship is a bilateral relationship.
8. Enmity is a bilateral relationship.

Rules 1–6 were applied because small children sometimes forgot to mention their kinship ties. Both friendship and enmity relationships were considered reciprocal which is why the analysis was focused on bilateral relationships, that is, the cases in which both students said they were friends (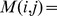
‘F’ and 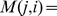
‘F’) or enemies (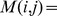
‘E’ and 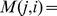
‘E’). Kinship relationships were included as friendship relationships. Therefore, by applying rules 1–8 the adjacency matrices become symmetrical matrices.

### Friendship Networks Analysis

**Figure 1:**
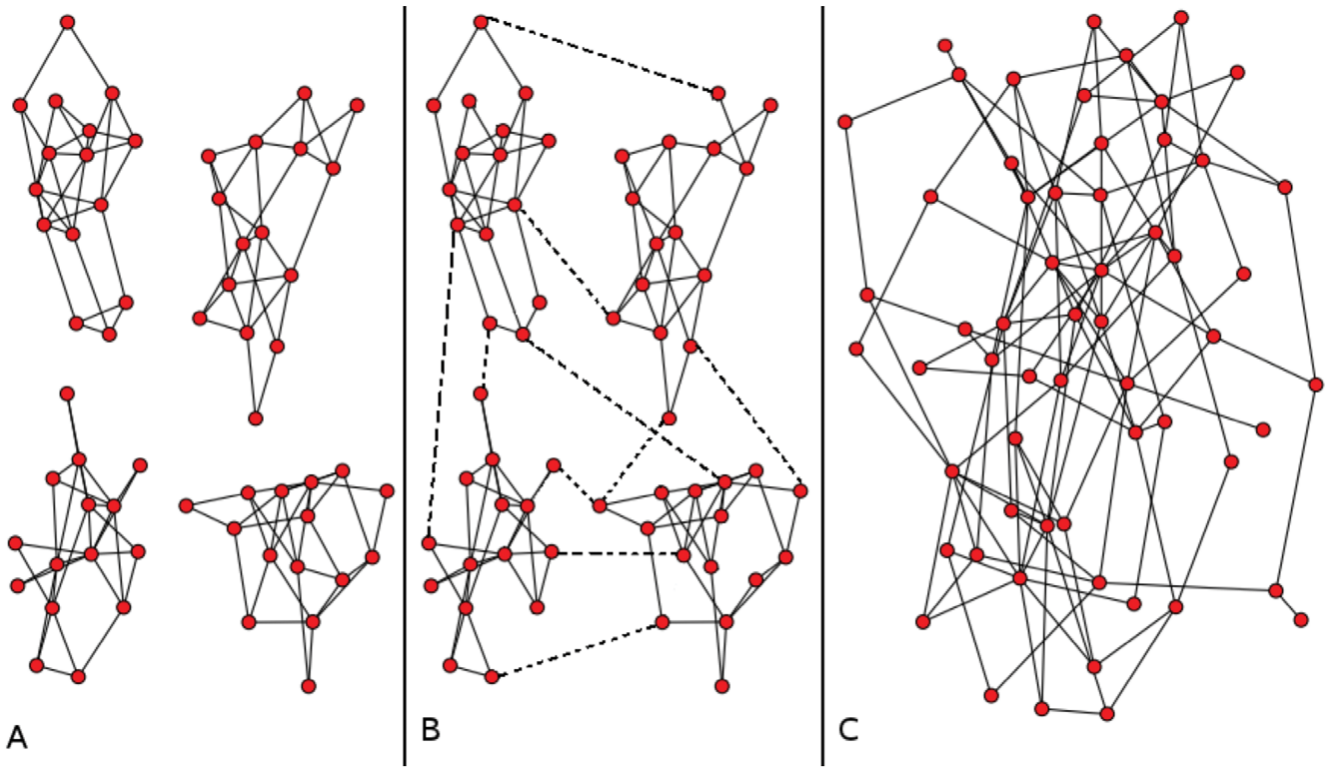
Friendship network topology. Figure 1 is a graphic representation of the friendship network topology, each node represents a student and lines between nodes indicate friendship relations. This example with 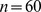, 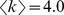 and 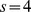. A) 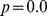; B) 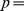, dashed lines represent introduced shortcuts; and C) 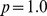.

Once the symmetrical matrices were generated, a friendship network was created for each studied school and their measurements calculated (all defined in [17]). All three school friendship networks shared the same properties (Table 1): 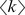 (average friends per student) was relatively high in all; they were low density networks; they had short path lengths; and a high clustering index. This similarity carried through when they were graphed (Fig. 1B).

**Table 1:** Friendship network measurements.

	School1	School2	School3
Order	108	226	419
Size	503	985	1575
<*k*>	9.315	8.717	7.518
Diameter	5	6	7
Density	0.087	0.039	0.018
Clustering	0.292	0.248	0.226
Geodesic	2.477	2.962	3.898
Betweenness	0.014	0.009	0.007
Closeness	0.407	0.340	0.259

Table 1 shows friendship network measurements at the three studied schools. We are using the Vega-Redondo notation [17] for the measurements of the network. In those cases where the definition applies to a single node we take the average over the complete network.

**Figure 2:**
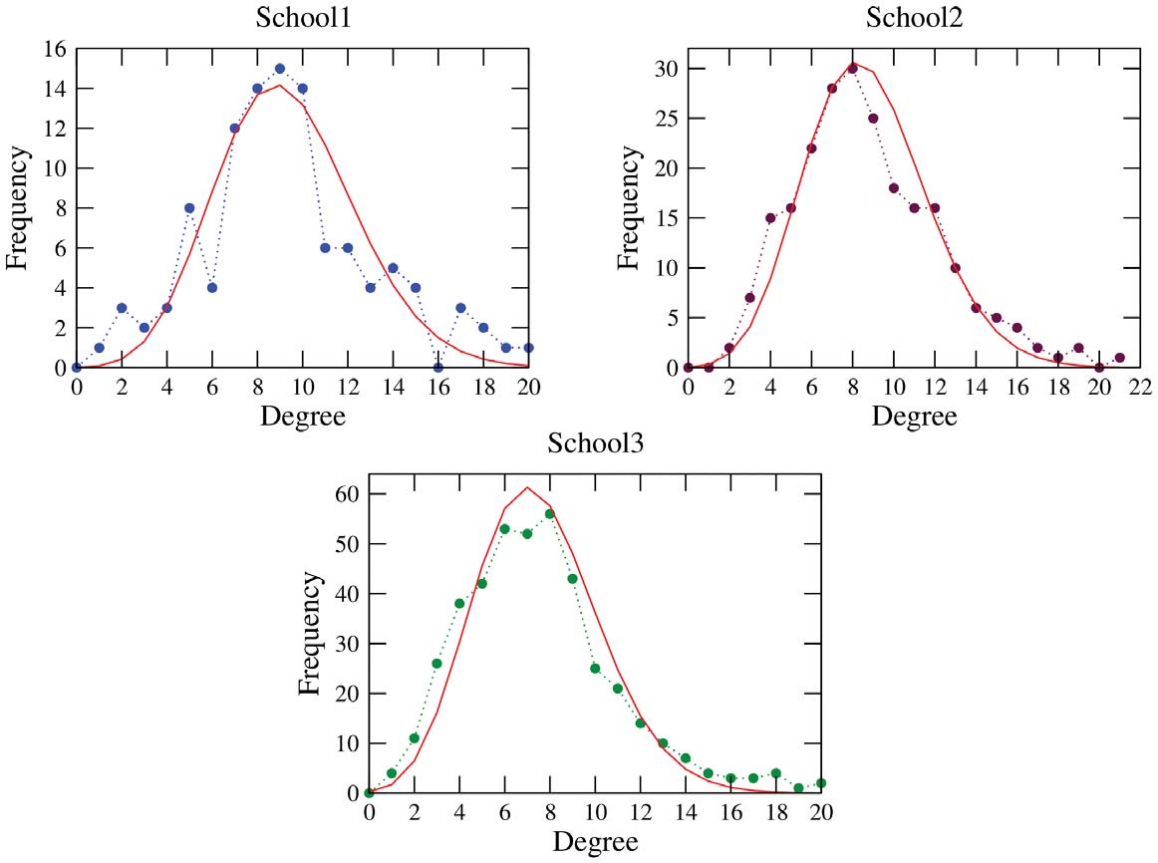
Friendship network degree distributions. Figure 2 shows degree distribution of friendship networks. Dots: Observed school friendship network degree distributions. Line: Adjusted Poisson(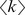) distribution (where 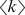 is the maximum likelihood estimator of the goodness of fit tests), with 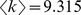, 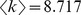 and 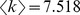 for School1, School2 and School3, respectively.

When the degree distributions were graphed for each school, we believed that they belonged to Poisson distributions (Fig. 2).

To verify that the friendship networks’ degree distribution originated in a Poisson distribution, we ran goodness-of-fit tests [18]. A Karl-Pearson statistic [19] was used to measure statistical differences between the observed data and the theoretical distribution (i.e. Poisson). For each test, 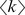 was the maximum likelihood estimator and in all three cases the p-value was sufficiently significant (Table 2), and therefore evidence exists that the friendship network distributions originated in a Poisson(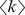) distribution; see the adjusted Poisson distributions (Fig. 2).

**Table 2:** 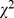
 test results.

	School1	School2	School3
<*k*>	9.315	8.717	7.518
*χ* ^2^	16.997	20.441	24.703
p-value	0.107	0.059	0.025

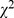
 test results, we prove that observed data meet a Poisson(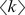) distribution, where 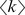 is the maximum likelihood estimator.

Given the Poisson distribution of the friendship networks at the three schools, it can be expected that they could be reproduced with the Erdös-Rényi (ER) model [20] because this generates complex networks with a degree distribution given by.
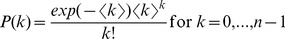
(2)


The ER model has two parameters: 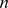 is the network order; and 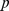 is the probability that a link exists between any 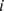 and 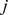 node pair. However, the clustering index will be low because in ER networks the clustering index (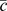) tends to be equal to network density. In the studied system, this means that the model did not reproduce the fact that if 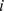 and 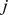 have a common friend in a certain student then 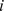 and 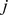 tend to be friends also.

The Watts-Strogatz (WS) model [21] is known to produce networks with high clustering index values. This model has three parameters: 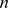 is the network order; 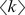 is the degree of the initial regular network; and 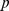 is the probability of redirecting each network link. However, the degree of distribution for WS networks, developed in [22], is given by.
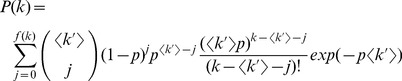
(3)and defined by 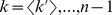, with 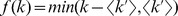 and 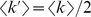. On the one hand, distribution 3 differs significantly from the Poisson distribution in that it tends to centralize in 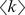. In the present study system, this is equivalent to saying that almost all the students would have 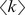 friends, thus leaving out introvert (few friendship relationships) and extrovert (many friendship relationships) students. On the other hand, distribution 3 tends toward a Poisson distribution when parameter 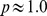, but when this occurs the model tends toward an ER model and, as mentioned previously, the ER model does not reproduce all observed measurements.

### Friendship Network Model

Neither the ER nor the WS models completely reproduced the friendship networks at the three studied schools. In response, we decided to develop a model to more accurately represent them. All three friendship networks exhibited spatial confinement caused by the fact that in schools students are grouped by classroom which is where they primarily interact. In other words, a student in a given classroom (e.g. 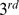 grade) has lots of friends in his classroom but few in other classrooms. In addition, students also experience a sense of belonging to a social group [23], [24]. These phenomena cause friendship networks to exhibit the atypical characteristic of a Poisson topology coupled with a high clustering index.

Spatial confinement and a sense of belonging are significant phenomena in these networks and were thus considered when designing the proposed model. What we call the School Friendship Network (SFN) encompasses four parameters: 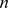, number of students; 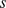, number of classrooms in the school; 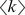, average number of friends per student; and 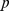, the probability of introducing shortcuts into the network. The goal was for the SFN model to reproduce the degree distribution and measurements observed in the three studied schools.

### Spatial Confinement and Sense of Belonging to a Social Group

To reproduce spatial confinement, it was decided to generate 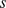 isolated networks 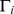 for 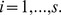, where each 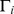 has the probabilistic construction ER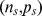 representing the friendship relationships within each classroom (Fig. 1A). In this way, 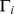 has 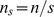 students (nodes) and a degree distribution as follows.
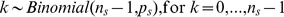
(4)where 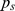 is the probability of any two students in the same classroom being friends. Given that in the ER networks 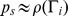 (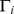 density) and 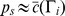 (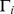 clustering index) are met, then from the first property follows



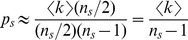
(5)Given that the entire network, called 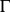, is defined by 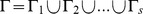, then the degree distribution is also given by distribution 4, and its clustering index, denoted 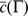, is given by.
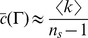
(6)


Equation 6 indicates that 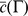 depends on 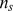 and 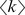 such that when classrooms are sufficiently large with respect to 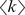, 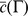 will be small. This assumes a problem in model construction. However, group dynamic theory [23] describes two types of groups: primary [25], and secondary [26]. Primary groups are composed of a small number of members with affective and intimately bonded relationships which share interests, values, goals, etc., and each member has a sense of belonging to the group. Secondary groups, in contrast, have a large number of members, which precludes proximity amongst them and any proximity is generally imposed (e.g. by institutional rules). In the relationship between these two group types, primary groups tend to appear within secondary groups. Taking this into account, we considered that relatively small classrooms have primary group characteristics, that is, members have a sense of belonging to the social group where they are spatially confined. If the group is large, however, it will have secondary group characteristics with primary groups forming within it which then interact inside the classroom in which they are spatially confined. We use this to apply a rule that will allow creation of subgroups within classrooms. Of note is that the social phenomenon of primary group formation within large classrooms also occurs at the studied schools, although it is not as evident as spatial confinement. We refer here to the fact that the spatial confinement produced by grouping into classrooms is evident in the adjacency matrices, but grouping within the classrooms produced by sense of belonging to a primary group is only evident in detailed observation of the interaction networks.

Based on the observed clustering indices, we proposed a threshold such that if 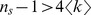, therefore, instead of creating 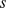 networks, 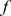 subnetworks are created within each 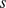 classroom, where 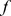 is given by 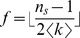
(7)


By applying this process, 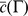 will be within the interval 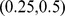 and the total network will be 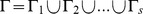, with 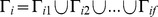 for 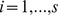. To create each one of the 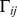 networks, a recursive process was applied which is analogous to that described previously in this subsection.

### Adding Shortcuts

The function 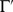 = shortcuts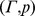 receives two parameters, where 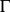 is a network composed of isolated subnetworks and 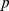 is the probability of creating shortcuts in 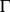 network. This is done by eliminating each 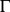 link with the probability 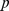, thus creating a new link between two randomly chosen 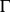 nodes. This process creates a 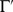 network which conserves the same number of nodes and links as 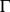 (Fig. 1B).

### Algorithm

The algorithm for the SFN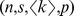 model involves four steps:

1. Calculate 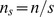, where 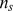 is the number of students per classroom.
2. If 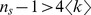:
  - Calculate 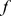 of Equation 7, where 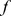 is the number of subnetworks for each classroom.
  - Calculate 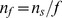 and 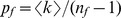, where 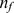 is the number of students in each subnetwork (within each classroom) and 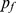 is the probability that any two students in the same subnetwork (within each classroom) will be friends.
  - For each of 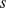 classrooms: Create 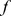 ER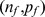 networks.
  - For each of 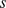 classrooms: Apply the shortcuts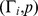 function, where each 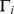 is the network formed by 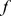 isolated networks.
3. If 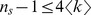:
  - Calculate 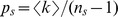, where 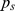 is the probability that any two students in the same classroom will be friends.
  - For each of 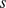 classrooms: Create an ER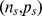 network.
4. Apply the shortcuts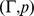 function, where 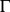 is the network formed by 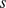 isolated networks.

In this way the model defines networks that are an interpolation between totally random isolated networks with a binomial distribution (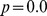) and a totally random network with a Poisson distribution (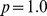) (Fig. 1). Each of the subnetworks (Fig. 1A), as well as the overall network (Fig. 1C), have the same probabilistic construction. Of note is that parameter 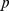 was expressly introduced, and a future research goal is to find a theoretical way of calculating 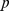.

**Figure 3:**
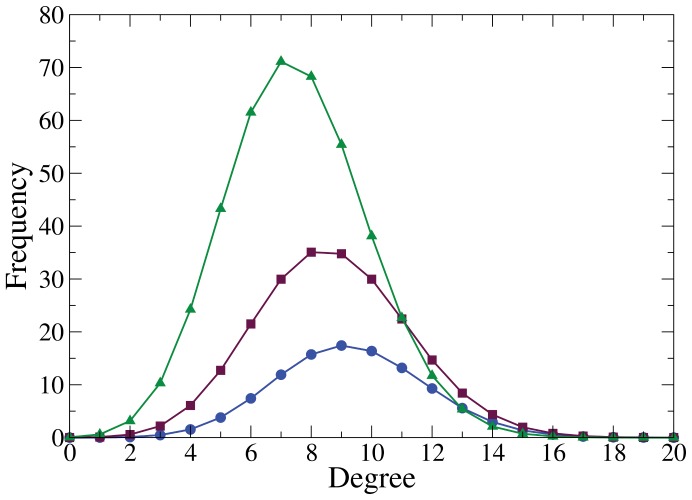
Friendship network degree distribution with SFN model. Figure 3 shows degree distribution of friendship networks with SFN model. Circles: 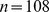, 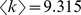, 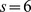 and 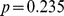. Squares: 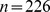, 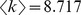, 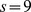 and 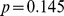. Triangles: 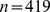, 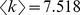, 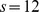 and 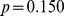. Each point corresponds to an average over 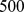 independent simulations.

When the SFN model was applied to the data, measurements (Table 3) and distributions (Fig. 3) did not differ from those observed in the studied schools. We ran Kolmogorov-Smirnov tests [18] to verify that the distributions produced by the SFN model did not differ statistically from the observed distribution (i.e. null hypothesis). The resulting p-values (School1 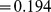; School2 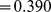; School3 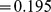) indicate that there is enough evidence to confirm that the distributions generated by the SFN model did not differ significantly from the empirical distributions.

**Table 3:** Friendship network measurements generated with SFN model.

	SFN1	SFN2	SFN3
Order	108	226	419
Size	502.84	985.13	1574.8
<*k*>	9.312	8.718	7.517
Diameter	4.684	5.913	6.918
Density	0.087	0.039	0.0180
Clustering	0.284	0.239	0.221
Geodesic	2.550	3.211	3.832
Betweenness	0.014	0.010	0.007
Closeness	0.395	0.315	0.261

Friendship network measurements generated with the proposed model. SFN1: 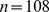, 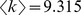, 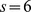 and 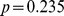. SFN2: 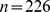, 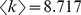, 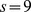 and 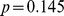. SFN3: 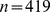, 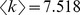, 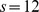 and 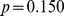. Each value corresponds to an average over 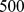 independent simulations.

Studies do exist of friendship networks in a school environment [2], [27] (e.g. Zachary karate club [28], college football [2]), but these are aimed at developing models to detect communities. The SFN model creates communities to produce a structure similar to the observed networks, with the same approximate measures and distributions.

### Enmity Network Analysis

**Figure 4:**
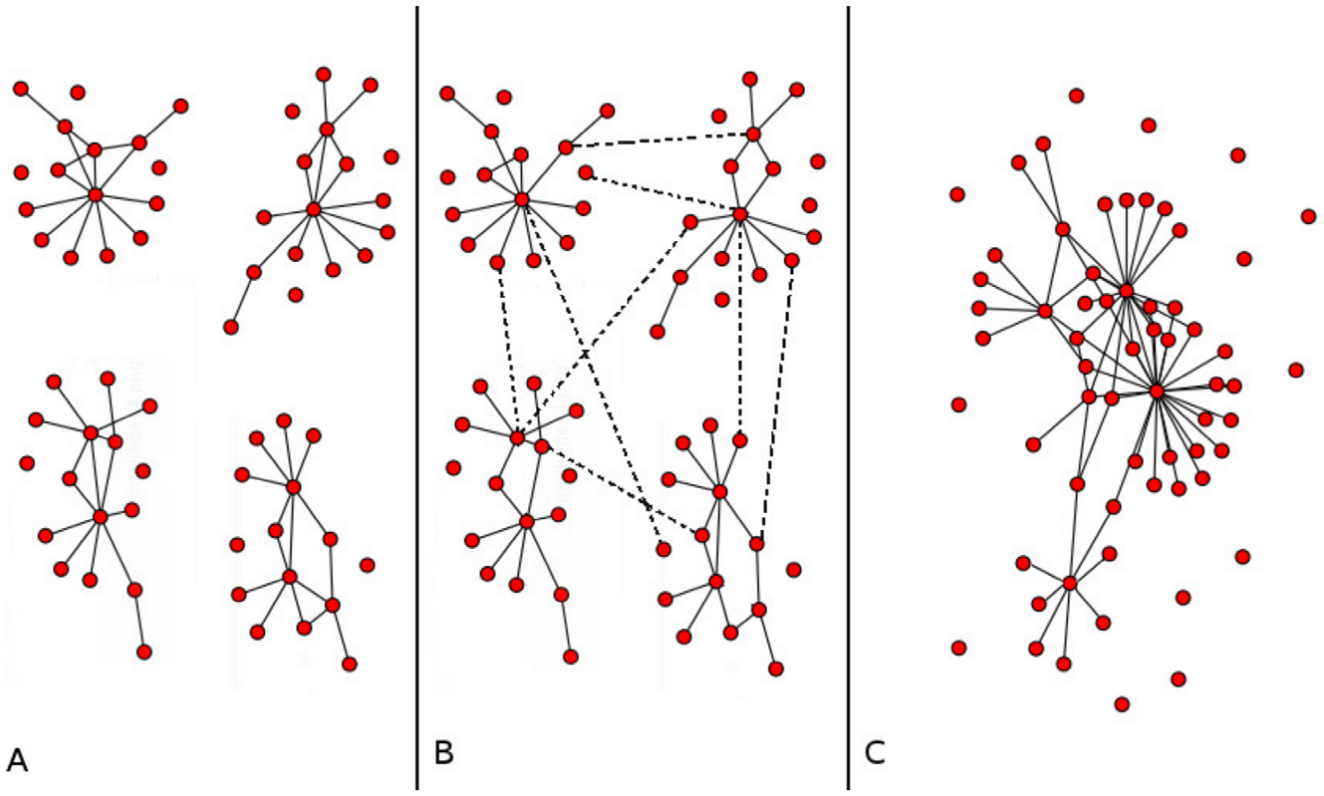
Enmity network topology. Figure 4 is a graphic representation of the enmity network topology, each node represents a student and lines between nodes indicate enmity relations. This example with 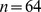, 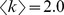 and 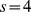. A) 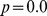; B) 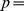, dashed lines represent introduce shortcuts; and C) 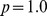.

**Figure 5:**
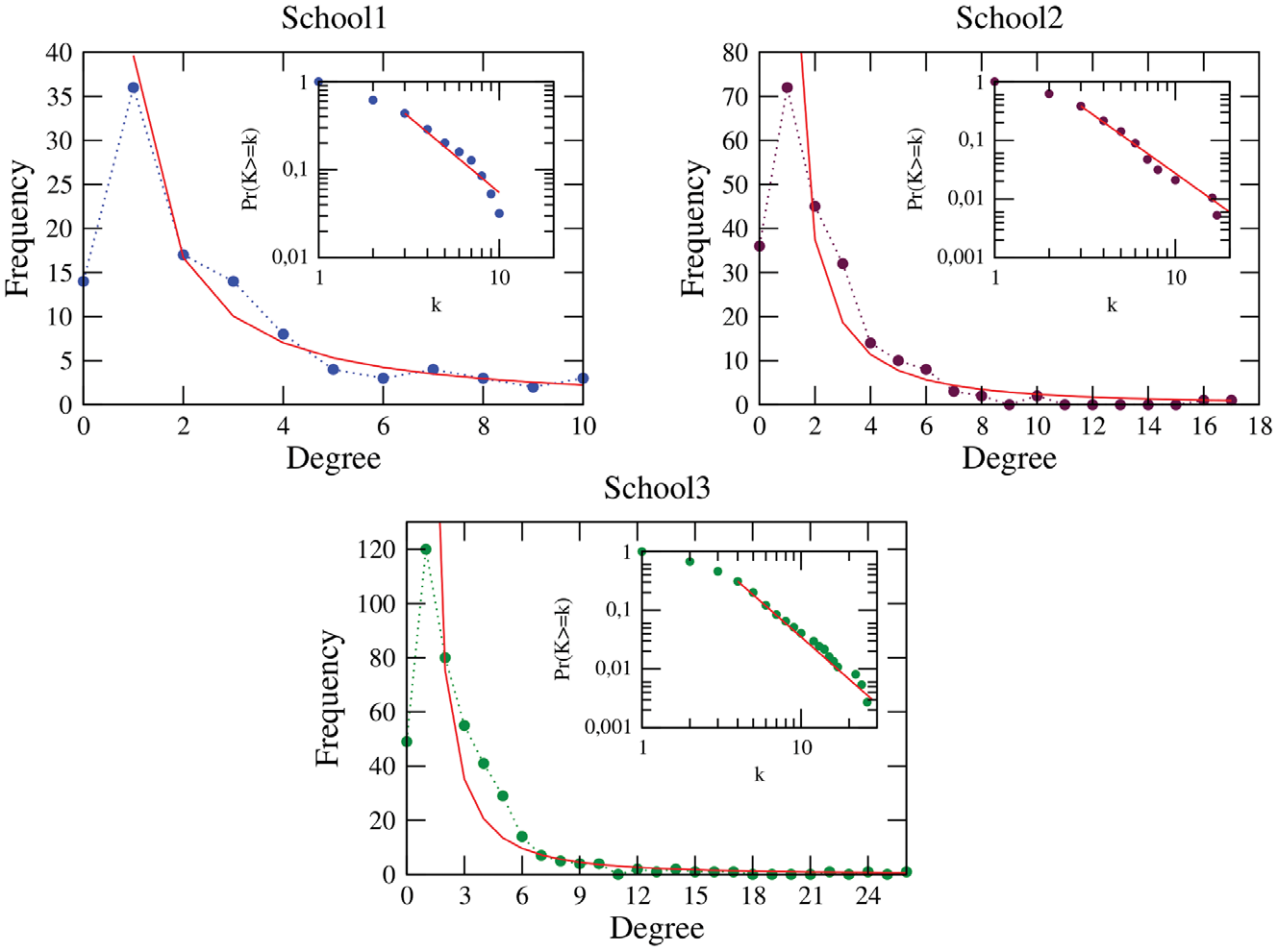
Enmity network degree distributions. Figure 5 shows degree distribution of enmity networks. Dots: Observed school enmity network degree distributions. Line: Potential regression model 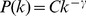, with 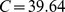, 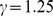; 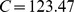, 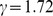 and 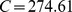, 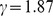 for School1, School2 and School3, respectively. Inset: Kolmogorov-Smirnov test, observed data and adjusted power law.

Among the three studied schools, the enmity networks had similar measurements; for example, all three had low 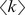 (average enemies per student) values, were low density networks, had short path lengths and a low clustering index value (Table 4). Since the three networks happen to be not connected, the diameter and betweenness are calculated from the principal component, while geodesic was estimated as the reciprocal of closeness. All three networks also had a similar structure when graphed (Fig. 4B). Degree distribution for the three schools (School1, School2, School3) was believed to conform to power law distributions (Fig. 5).

**Table 4:** Enmity network measurements.

	School1	School2	School3
Order	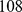	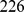	419
Size	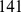	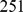	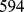
<*k*>	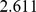	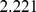	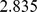
Diameter	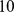	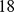	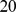
Density	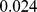	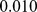	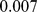
Clustering	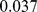	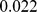	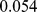
Geodesic	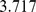	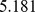	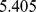
Betweenness	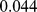	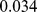	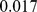
Closeness	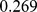	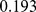	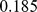

Table 4 shows enmity network measurements in the three studied schools. We are using the Vega-Redondo notation [17] for the measurements of the network. In those cases where the definition applies to a single node we take the average over the complete network.

To verify that the enmity networks degree distributions originated in a power law distribution, potential regression tests [29] were done with the model 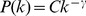. This was done without including 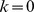. The 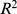-adjusted was greater than 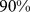 in all three cases (Table 5), although this test is inconclusive, only suggesting that the observed data could be distributed under a power law.

**Table 5:** Potential regression model adjustment and Kolmogorov-Smirnov test results.

	School1	School2	School3
γ	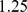	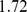	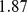
*C*	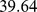	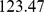	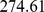
*R* ^2^-adjusted	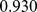	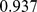	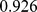
α	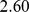	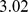	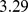
*k_min_*	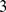	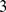	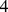
*p*-value	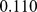	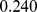	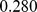

Top: Potential regression model adjustment, 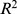-adjusted value show fit greater than 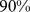 in all three cases. Bottom: Kolmogorov-Smirnov test results, we prove that observed data meet a distribution in equation 8, where 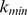 is the lowest 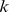 value for which the power distribution hold and 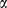 is the maximum likelihood estimator.

Kolmogorov-Smirnov tests, described in [30], were run to improve validation, adjusting the data to the distributions
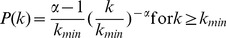
(8)where 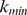 is the lowest 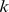 value for which the power distribution is met and 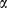 is the maximum likelihood estimator for the observed data; both were estimated as described in [30]. This test uses the 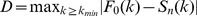 statistic, which measures the maximum absolute difference of the accumulated distribution functions for the observed data and theoretical distribution. In all three cases, the p-value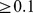, and therefore evidence exists that the enmity network distribution tails originated in a power law (Table 5). This is visible in the graphics showing the observed data and corresponding adjusted power law for each school (Fig. 5).

Once it was clear that the enmity networks exhibited a distribution with a power law tail, it is to be expected that the Barabási-Albert (BA) model [31] could reproduce them. This model has a distribution given by
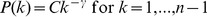
(9)where 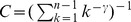 and 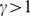. There are two parameters in the BA model: 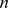 is the network order, and 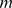 is the number of links contributed by each node as it enters the network. However, this model produces networks in which all nodes are at least 1 degree and are connected. This means that these networks’ degree distributions differed significantly from those of the studied enmity networks.

### Enmity Network Model

Once it was confirmed that the enmity networks were not completely reproduced by the BA model, we decided to develop a model to more accurately represent them. Distributions exist with this form.
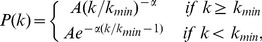
(10)where 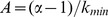 [30]. Based on the previous tests and the Fig. 5 (graphics inset), we conclude that distribution 10 best represents the observed data. This being the case, the proposed model must contemplate both preferential attachment (to model the power law) and randomness (to model the exponential) when links are introduced into the network. As is to be expected, spatial confinement also occurs in the studied enmity networks, which is why the School Enmity Network (SEN) includes four parameters: 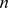 is number of students; 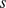 is number of classrooms; 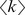 is average number of enemies per student; and 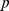 is the probability of introducing shortcuts into the networks. In contrast to the SFN model, the SEN model generates networks with preferential attachment, applying the rules.
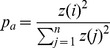
(11)where 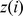 is the degree of node 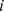, that is, a node has a greater probability of being selected when its degree is higher. The SEN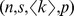 model algorithm is as follows.

1. Calculate 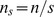 and 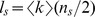, where 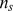 is number of students per classroom and 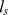 is number of enmity relationships per classroom.
2. For each one of 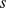 classrooms: Create a 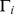 network (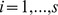) with preferential attachment, where 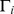 will have 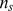 nodes and 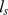 links introduced by connecting two of its nodes, one chosen preferentially according to equation 11 and the other chosen randomly (Fig. 4A).
3. For each link in the 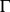 network (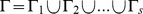), this is eliminated with probability 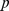 and a new link created between two 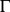 nodes (one chosen preferentially according to equation 11 and the other chosen randomly).

**Figure 6:**
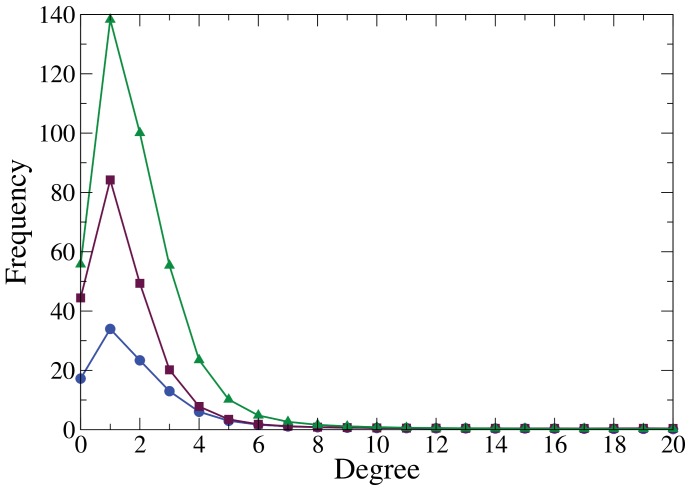
Enmity network degree distribution with SEN model. Figure 6 shows degree distribution of enmity networks with SEN model. Circles: 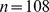, 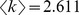, 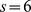 and 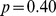. Squares: 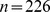, 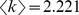, 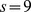 and 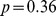. Triangles: 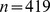, 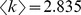, 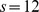 and 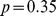. Each point corresponds to an average over 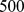 independent simulations.

Therefore, the model defines networks which are an interpolation between isolated preferential attachment networks (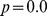) and a preferential attachment network (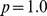) (Fig. 4). As occurred with the SFN model, each of the subnetworks (Fig. 4A) and the overall network (Fig. 4C) had the same probabilistic construction. Measurements were then generated by applying this model to the observed data (Table 6), and degree distributions for these networks graphed (Fig. 6). We ran Kolmogorov-Smirnov tests comparing the SEN model distributions with the observed distributions. The resulting p-values (School1 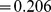, School2 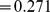, School3 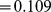) indicate that these distributions do not differ significantly from the empirical values.

**Table 6:** Enmity network measurements generated with SEN model.

	SEN1	SEN2	SEN3
Order	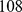	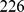	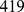
Size	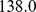	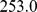	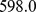
<*k*>	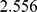	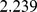	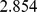
Diameter	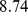	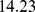	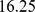
Density	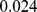	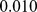	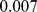
Clustering	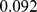	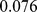	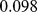
Geodesic	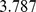	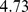	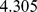
Betweenness	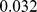	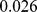	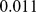
Closeness	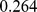	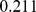	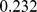

Enmity network measurements generated with the proposed model. SEN1: 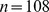, 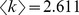, 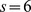 and 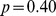. SEN2: 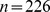, 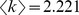, 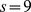 and 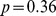. SEN3: 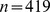, 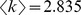, 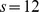 and 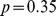. Each value corresponds to an average over 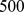 independent simulations.

Models do exist which are more flexible in response to the introduction of links into the network (e.g. extended BA model [32]). Depending on their parameters, they can generate networks with distribution 10, even though these do not consider the spatial confinement, a characteristic vital to reproducing the structure of the networks we are studying.

## Conclusions and Discussion

In the three studied schools, friendship relationships had a Poisson topology while enmity relationships had a power law topology. New models were necessary to accurately reproduce the observed data, both in terms of measurements and degree distributions. Spatial confinement and a sense of belonging to a social group both played important roles since their incorporation allowed studying and understanding the characteristics and phenomena which occur in the studied school networks.

As mentioned in section ‘Methodology’, School1 had one classroom containing two grades (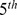 and 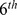). For study purposes, these groups were treated as separate classrooms because the principle observed in subsection ‘Spatial confinement and sense of belonging to a social group’ was observed here. Despite the spatial confinement in this classroom, the sense of belonging to a primary group was manifested. In response, two subgroups were created, one of 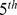 grade students and the other of 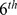 grade students, with some interactions between them, exactly as if they were two classrooms.

Promising future research areas include theoretical analysis of the network properties produced in these models. Another possible study would be to apply a diffusion process (e.g. disease transmission) to these networks, observe how the disease infects other students and propose ways of preventing propagation. An analysis could also be run of the link(s) between the friendship network and enmity network within the same school. Another interesting area of inquiry is network assortativity classes [33], that is, the tendency observed in social networks in which nodes connect to other nodes with similar properties. This property generally refers to the degree of nodes, but we can also speak of social assortativity (as mentioned previously) in the studied friendship and enmity networks. Assortativity manifests in our model because students mainly relate to students in their own classroom. After analyzing the networks, however, other types of assortativity become evident, such as sex, in which boys have friendships and enmities mainly with boys and girls mainly with girls. In rural schools, assortativity occurs based on kinship in that students have friendships with relatives, although this does not hold for enmity networks.

The proposed models (SFN and SEN) generated complex networks with fractal characteristics. It is highly probable that a study of the friendship and enmity networks between students from different schools in the same location would find that the relationships between schools have the same structure as the relationships observed here between classrooms. In other words, there would be a high number of relationships between students at the same school and few between students from different schools. This pattern could repeat itself in an analysis of relationships between students from different locations, thus forming a fractal structure. If this were the case, the proposed models could be generalized and used to represent the network structure of an entire community, although reaching this point will require further research.
