# Correction: Modeling Social Network Topologies in Elementary Schools

**DOI:** 10.1371/annotation/235b0944-47f4-4d11-9472-5715d14cf43f

**Published:** 2013-09-24

**Authors:** Rodrigo Huerta-Quintanilla, Efrain Canto-Lugo, Dolores Viga-de Alva

A number of errors were introduced into the figure legends during preparation of this article for publication. In figure legends 1 and 4, part B) should read: B) p=ε, dashed lines represent introduced shortcuts

Additional formatting errors were introduced in equations 2, 4 and 8. In each of these equations there are missing spaces surrounding the word "for." 

